# Can Satellite Remote Sensing Assist in the Characterization of Yeasts Related to Biogeographical Origin?

**DOI:** 10.3390/s23042059

**Published:** 2023-02-11

**Authors:** David Castrillo, Pilar Blanco, Sergio Vélez

**Affiliations:** 1 Estación de Viticultura e Enoloxía de Galicia (EVEGA-AGACAL), Ponte San Clodio s/n, 32428 Leiro-Ourense, Spain; 2 Information Technology Group, Wageningen University & Research, 6708 Wageningen, The Netherlands

**Keywords:** NDVI, Designation of Origin, vegetation management, precision agriculture, organic production, microbial terroir, satellite sensors, Sentinel, Landsat, normalized difference index of vegetation

## Abstract

Biogeography is a key concept associated with microbial terroir, which is responsible for the differentiation and uniqueness of wines. One of the factors influencing this microbial terroir is the vegetation, which in turn is influenced by climate, soil, and cultural practices. Remote sensing instruments can provide useful information about vegetation. This study analyses the relationship between NDVI, calculated using Sentinel-2 and Landsat-8 satellite images of different veraison dates, and microbial data obtained in 2015 from 14 commercial (organic and conventional) vineyards belonging to four Designations of Origin (DOs) from Galicia (northwest Spain). Microbial populations in grapes and musts were identified using PCR techniques and confirmed by sequencing. Statistical analyses were made using PCA, CCA, TB-PLS, and correlation analyses. This study confirms that the NDVI is positively correlated with the diversity of yeasts, both in grapes’ surface and must samples. Moreover, the results of this study show: (i) Sentinel-2 images, as well as Landsat-8 images, can establish differences in NDVI related to yeast terroir in grapes and musts, as it is the most relevant DO factor, (ii) Sentinel-2 NDVI and yeast biogeography are moderately to strongly correlated, (iii) Sentinel-2 achieved a better delimitation of the DOs than Landsat-8 and can establish more accurate differences in NDVI–yeast terroir correlations, and (iv) a higher NDVI was associated with the yeast biogeographical patterns of the DOs with higher species richness (S) consisting of weakly fermenting yeasts (*Hanseniaspora uvarum, Pichia* spp., *Starmerella bacillaris,* and *Zygosaccharomyces* spp). However, NDVI values did not correlate well with biogeographic patterns of yeasts previously studied at frequency level (proportion or percentage of each species) in each particular DO. This study suggests that satellite imagery has the potential to be a valuable tool for wine quality management and a decision-making instrument for DO regulators and winegrowers.

## 1. Introduction

Terroir is one of the most important concepts in wine [1]. Although the varieties of grapes grown in a particular region of the world are essentially determined by soil and its average climatic conditions, with climate variations and trends influencing year-to-year variations, viticultural and enological techniques can unquestionably be linked to wine quality [2]. The term “terroir” involves all these components, and the Designation of Origin (DO) system is based on this premise. It describes how the local environment, agricultural methods, crop features, and cultural aspects interact in a certain way that cannot be replicated in any other region to influence the growth and production of grapes and wine. In Europe, it is also known as *Appellation d’Origine Protégée* (AOP, French), and *Denominación de Origen Protegida* (DOP, Spanish). This concept assumes that the factors that affect a plant’s environment, such as the soil, the microbiome, or the climate, confer a particularly distinctive character to that growing place [3].

On the one hand, remote sensing has already demonstrated its potential application in large-scale agriculture [4]. Imagery may be gathered from many sources, including satellites, aeroplanes, proximate sensing, and UAVs (unmanned aerial vehicles). Each strategy has benefits and drawbacks. Satellite data can help compare vineyards within the same and different DOs, as it can simultaneously capture a massive image of the whole region. Additionally, satellites are unrepairable once launched, so their sensors typically have superior electronics and redundancy, as well as greater spectral and radiometric resolution [5]. Even a breakpoint set at somewhat more than 5 ha suggests that above such scale sizes, satellite photos may be more useful than other platforms [6]. Some satellite images, such as Sentinel-2 and Landsat-8 imagery, are also available for free download. Landsat-8 has the OLI instrument, a multispectral sensor with a resolution of 30 m that gives an image every 16 days [7]. Sentinel-2 is equipped with another multispectral sensor, the MSI instrument, with a resolution of 10 m that can provide an image every ten days. In addition, it is possible to obtain an image every five days by integrating the data from the two spacecraft belonging to the Sentinel-2 constellation. Both satellites offer a variety of bands from which to obtain data, allowing the calculation of vegetation indices (VIs), such as NDVI (normalized difference of vegetation index) [8]. NDVI measures the relationship between red and NIR (near infrared) bands and can be a useful tool for vineyard analysis [9,10], monitoring the quality characteristics [11], or differentiating zones according to the vegetation [12]. In addition, satellite-derived NDVI has already been linked to vineyard quality assessment [13], helping to design sampling protocols for monitoring grape ripening [14].

On the other hand, one of the pillars in the differentiation of wines associated with a given terroir is the biogeographical patterns of the microbiota, and especially the indigenous yeasts present in each vineyard, as generators of added value and distinction in the wine of a given region [15]. In a previous three-year study, we showed how NDVI calculated from satellite imagery, which is related to vegetation, may be applied to determine differences in terroir and yeast microbiota [16]. Besides, a comprehensive characterisation of indigenous non-*Saccharomyces* yeast strains with potential use in winemaking was conducted in other works [17,18,19,20].

Consequently, we defined the following objectives: (i) analyse the relationship between Landsat-8 and Sentinel-2 satellite imagery (NDVI) and yeast diversity, including richness and frequency of yeast species in grapes and musts, and (ii) compare previously found yeast biogeographical patterns [18] to satellite NDVI values according to the DO.

## 2. Materials and Methods

### 2.1. Experimental Trial and Yeast Identification

**Figure 1:**
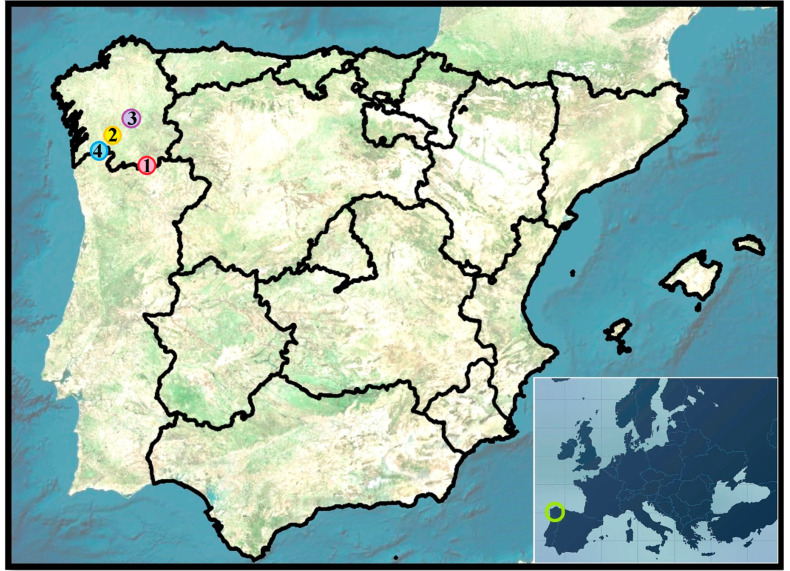
Location of the vineyards in Galicia (Spain). Climatic zones: Monterrei (1: point in red) zone II (indoor oceanic climate); Ribeira Sacra (3: point in purple) zone II-III (indoor—Mediterranean oceanic climate); Ribeiro (2: point in yellow) zone III (Mediterranean oceanic climate); Rías Baixas (4: point in blue) zone I (coastal oceanic climate). Bottom-right: green circle shows the location of Galicia region within Europe.

**Figure 2:**
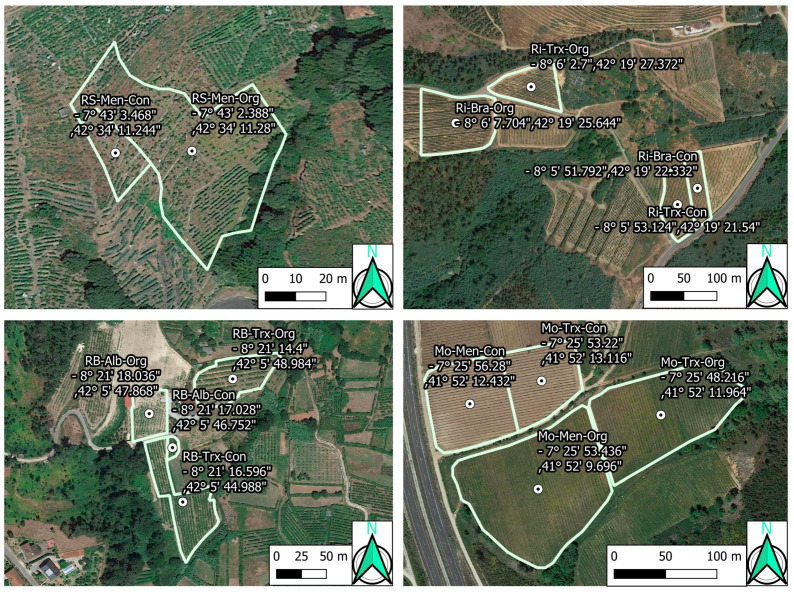
Vineyard locations and coordinates (WGS84). Designations of Origin (DOs: Monterrei, Mo; Rías Baixas, RB; Ribeira Sacra, RS; Ribeiro, Ri); culture system (Organic, Org; Conventional, Con); grapevine varieties (Albariño, Alb; Brancellao, Bra; Mencía, Men; Treixadura, Trx).

**Figure 3:**
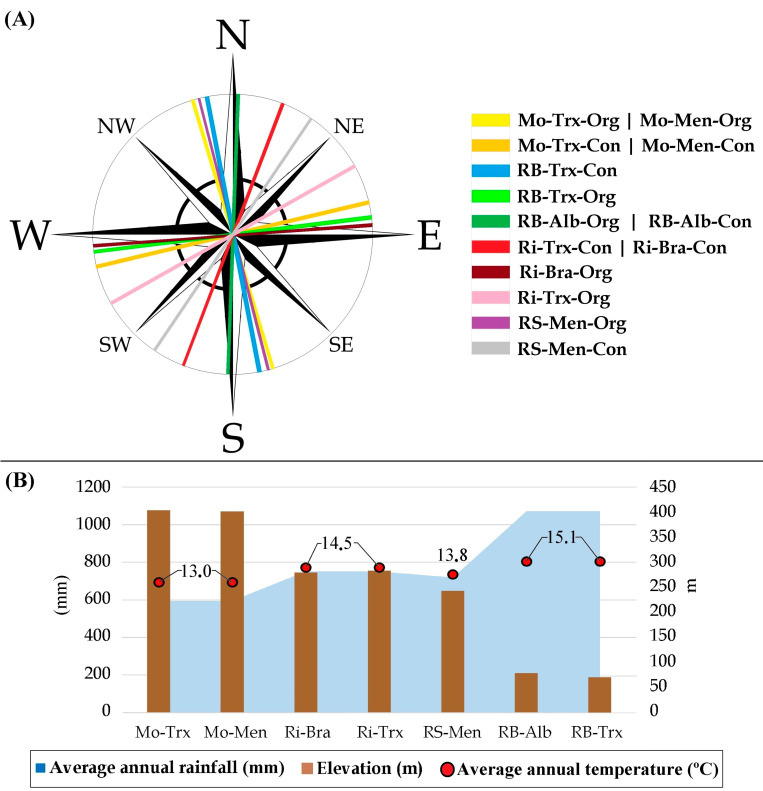
(**A**) Orientation and (**B**) elevation, average annual rainfall, and average annual temperature of each vineyard during 2015. Designations of Origin (DOs: Monterrei, Mo; Rías Baixas, RB; Ribeira Sacra, RS; Ribeiro, Ri); culture system (Organic, Org; Conventional, Con); grapevine varieties (Albariño, Alb; Brancellao, Bra; Mencía, Men; Treixadura, Trx).

Forty-two samples from fourteen organic and conventional commercial vineyards were collected in 2015. The vineyards were located within four different DOs (Monterrei, Ribeiro, Ribeira Sacra, and Rías Baixas) in Galicia, Spain (Figure 1 and Figure 2). Each vineyard was planted using grapevine cultivars representative of each DO. This way, Monterrei DO included four vineyards planted in vertical trellis with Treixadura (Mo-Trx) and Mencía (Mo-Men). Ribeiro DO included four vineyards planted in vertical trellis with Brancellao (Ri-Bra) and Treixadura (Ri-Trx). Rías Baixas DO included four vineyards planted in pergola trellis system with Albariño (RB-Alb) and Treixadura (RB-Trx). Finally, Ribeira Sacra DO included two vineyards planted without vegetation management with Mencía (RS-Men). The vineyards had different elevations, rainfall, and orientations ranging from N-S to E-W (Figure 3).

Finally, yeast diversity identification was carried out by isolating yeasts from healthy whole grapes and their juice, according to the procedures described in [18].

### 2.2. Sentinel-2 and Landsat-8 Multispectral Images

Sentinel-2 and Landsat-8 images from July and August 2015 were downloaded because the grapes ripened (veraison) on these dates. Several authors established that this phenological stage is an optimal time for modelling because the relationship with NDVI is more significant and the canopy may not be fully developed in earlier stages [21,22,23]. Moreover, NDVI has been used for many applications related to quality parameters in vineyards. Thus, this index has been used to estimate various parameters related to harvest suitability [24], to differentiate grape maturity [25], and to monitor the quality characteristics of table grapes [11]. Therefore, NDVI was selected as the vegetation index to perform the analysis.

Sentinel-2 and Landsat-8 were selected because they regularly provide free imagery. Landsat-8 provides moderate-resolution measurements every 16 days in the visible, thermal infrared, and near and shortwave infrared. Landsat-8 has two science instruments: the Operational Land Imager (OLI) and the Thermal InfraRed Sensor (TIRS), with a 15-metre-resolution panchromatic band, 30-metre-resolution visible, NIR, and SWIR bands, and 100-metre-resolution thermal band. Data from the OLI sensor were used for this study. On the other hand, Sentinel-2 is a pair of satellites that offer images every 5 days, operating in polar, sun-synchronous orbits. The two satellites occupy the same orbit but are 180 degrees apart and take ten days to complete one orbit; therefore, the temporal resolution of the images is five days for the two satellites combined. In addition, they mount the MSI sensor, which offers 13 spectral bands with a resolution of 10, 20, and 60 metres, in the visible, near-infrared, and shortwave infrared (ESA, 2015).

### 2.3. Data Analysis

R (v.4.2.X, R Core Team 2022, Vienna, Austria), including packages spatstat, raster, rgdal, sf, and rgeos, obtained from the Comprehensive R Archive Network (CRAN), and PAST software (v.4.07b 2021, Hammer, Harper & Ryan, Oslo, Norway) were employed for data analysis.

PERMANOVA was used to determine the influence of DO, cropping system and variety factors on NDVI, and yeast diversity at species richness level (S: number of different species) and frequency (proportion of each yeast species) in grapes and musts.

In addition, principal component analysis (PCA) and partial least squares (TB-PLS) were performed to separate NDVI, S, and yeast frequency. Furthermore, to assess the correspondence and correlation between NDVI and yeast diversity, a canonical correlation analysis (CCA) and a Pearson correlation were performed. In addition, to explore the possibilities of using NDVI to assess yeasts in vineyards, linear regression analyses were performed.

Finally, a Composite CCA was conducted to evaluate the impact of several factors that characterise terroir on NDVI and yeast diversity concerning the different DOs: farming systems (organic and conventional), canopy, vineyard orientation, elevation, and soil management. Moreover, monthly averages of relative humidity, wind speed, rainfall, and temperature were considered.

## 3. Results

### 3.1. Previous Exploratory Analysis

**Figure 4:**
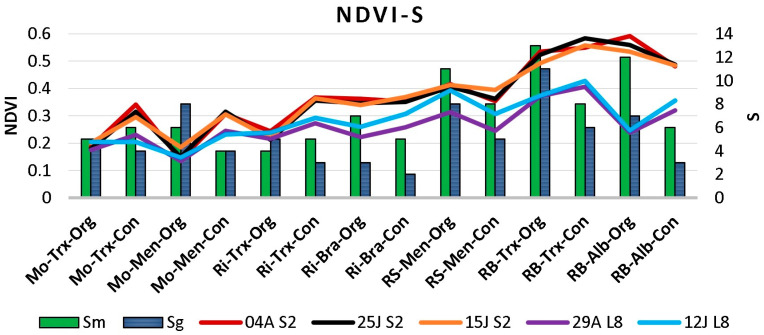
NDVI values and species richness in musts (Sm) and grapes (Sg) per DO (Monterrei, Mo; Rías Baixas, RB; Ribeira Sacra, RS; Ribeiro, Ri), cultivation system (Organic, Org; Conventional, Con), and cultivar (Albariño, Alb; Brancellao, Bra; Mencía, Men; Treixadura, Trx) calculated using Sentinel-2 (S2) and Landsat-8 (L8) imagery close to veraison (04A: 4 August; 25J: 25 July; 15J: 15 July; 29A: 29 August; 12J: 12 July).

The presence of clouds in several images made some dates unavailable. Figure 4 shows: (i) the species richness in musts and grapes in each DO, farming system, and grape variety; and (ii) the NDVI values calculated from cloud-free images captured with Sentinel-2 and Landsat-8 at various dates close to and after veraison. More detailed data on species richness and frequency of species can be found in Castrillo et al. [18].

Results show that satellite NDVI was related to S, with different NDVI values and S for each DO (Figure 4). Moreover, the values were lower in Ribeiro and Monterrei than in Rías Baixas and Ribeira Sacra DOs. The maximum values for each culture system can be observed in the Rías Baixas DO, particularly in Sentinel-2. When NDVI was analysed by culture system, no clear association or pattern was observed between organic and conventional, nor by grape variety.

Regarding the maximum NDVI, the highest values were found in Rías Baixas (0.59) and Ribeira Sacra (0.42), whereas the lowest were observed in Ribeiro (0.37) and Monterrei (0.34). On the contrary, for the minimum NDVI values, Monterrei showed the lowest value (0.13), followed by Ribeiro (0.21), and finally, Rías Baixas and Ribeira Sacra had the same value (0.24).

Depending on the vineyard and the satellite, the maximum NDVI values occurred between 15 July and 4 August. However, the minimum values are found on 29 August in almost every vineyard. Therefore, as a starting point, certain differences can be observed between the NDVI values of the Sentinel and Landsat images, and these differences are not uniform in all the DOs. Other authors have reported similar issues, where instruments mounted on different platforms and capturing the same area provide different values in images, showing significant inconsistencies [26,27].

Bray-Curtis PERMANOVA confirmed the above observations. The results obtained for Landsat-8 are detailed in [16], highlighting that the NDVI only showed significant differences between DOs. The results obtained for Sentinel-2 are shown in Table 1.

### 3.2. In-Depth Analysis of the Microbial Terroir

The relationship between the data provided by the images and the field data related to the yeast patterns in each vineyard was studied.

Bray-Curtis PERMANOVA (Table 1) showed significant differences (*p* < 0.01), and high F-values were found between NDVI–DO and DO–culture system combinations. Moreover, the pairwise RB–Mo and RB–Ri showed significant differences (*p* < 0.05). However, no significant differences were found between farming systems and grapevine varieties. ANOSIM and PERMANOVA also showed similar significant differences in yeast species diversity across the four DOs and cultivation systems [18].

**Figure 5:**
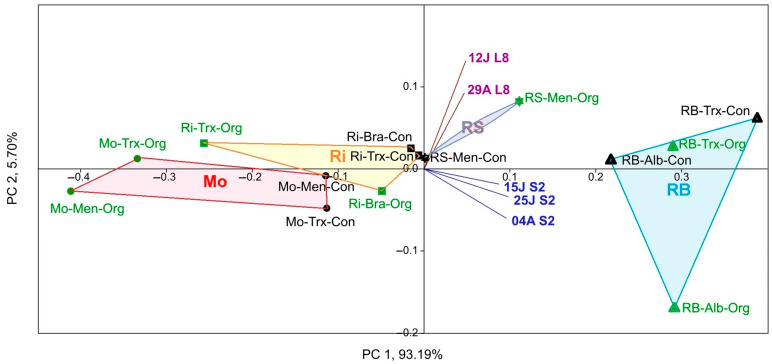
PCA of NDVI calculated using Sentinel-2 (S2 in dates 04A: 4 August, 25J: 25 July, and 15J: 15 July) and Landsat-8 (L8 in dates: 29A: 29 August and 12J: 12 July) per DO (circle: Monterrei, Mo; triangle: Rías Baixas, RB; asterisk: Ribeira Sacra, RS; square: Ribeiro, Ri), cultivation system (Organic, Org (green symbols); Conventional, Con (black symbols)) and cultivar (Albariño, Alb; Brancellao, Bra; Mencía, Men; Treixadura, Trx).

On the other hand, a PCA was used to group the different factors (DOs, culture system, and variety), possibly correlated, into a set of uncorrelated variable values, considering the Sentinel-2 and Landsat-8 NDVI (Figure 5). As a result, PCA separated Rías Baixas and Ribeira Sacra from Ribeiro and Monterrei DO samples on both sides of the Y-axis.

No significant differences were found in NDVI according to the farming system and cultivar factors. However, a slight gap was shown in NDVI values for the latter factor. In addition, the NDVI values of the two satellites were separated into two different quadrants, but both were directed towards the RS and RB DOs.

**Figure 6:**
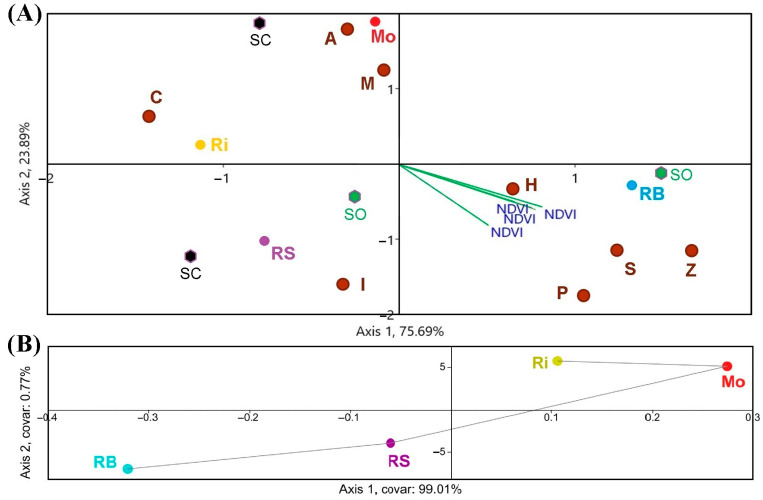
(**A**) CCA, (**B**) TB-PLS of the NDVI–species richness correlation. Single letters are the species (A, *Aureobasidium* spp.; C, *Cryptococcus* spp.; H, *Hanseniaspora uvarum*; It, *Issatchenkia terricola*; M, *Metschnikowia* spp.; P, *Pichia* spp.; S, *Starmerella bacillaris*; Z, *Zygosaccharomyces* spp.) in the four DO (Monterrei, Mo; Rías Baixas, RB; Ribeira Sacra, RS; Ribeiro, Ri), according to organic (SO) and conventional production system (SC).

**Figure 7:**
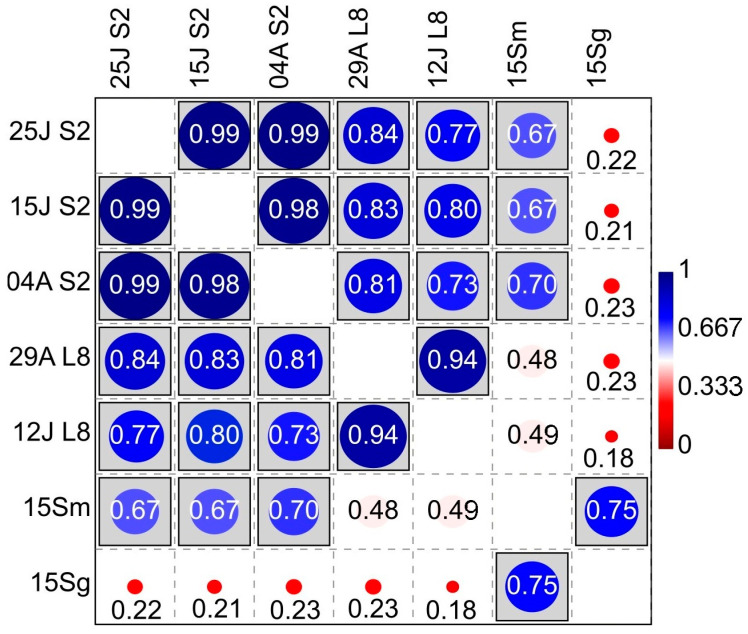
Correlations (Pearson’s r). Analysed factors: Sentinel-2 NDVI (S2), dates: 4 August, 25 July, and 15 July. Landsat-8 NDVI (L8), dates: 29 August and 12 July. Yeast species richness in grapes (15Sg) and musts (15Sm). Statistical ‘linear r (Pearson) and p(uncorr)’ table format: significance at *p* < 0.05 is boxed.

Figure 6 shows the conducted CCA and TB-PLS to find correspondences between NDVI and S. Furthermore, Figure 7 shows the NDVI-S correlations.

Regarding the CCA (Figure 6A), the NDVI values from the Sentinel-2 triplot were clustered towards the Rías Baixas DO, especially with the species richness corresponding to the organic vineyards. This way, Shannon-Wiener, Simpson’s diversity and equitability indices reveal that Rías Baixas had the highest biodiversity, followed by Ribeira Sacra. However, SIMPER analysis showed that the most prevalent species were *Aureobasidium* spp. *Hanseniaspora uvarum*, *Cryptococcus* spp. *Metschnikowia* spp., *Starmerella bacillaris*, *Lachancea thermotolerans,* and *Debaryomyces hansenii* [18]. Regarding the number of species, a higher NDVI value corresponded to *H. uvarum*, *Pichia* spp., *Starm. bacillaris* and *Zygosaccharomyces* spp. Both TB-PLS (Figure 6B) and PCA (Figure 5) were able to differentiate between DOs.

**Figure 8:**
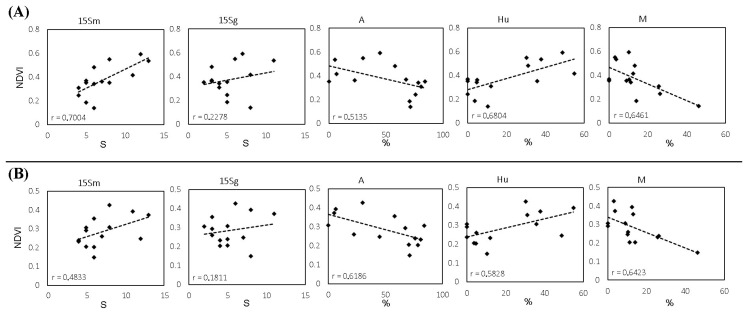
Linear regression analysis. (**A**) Sentinel-2 NDVI (4 August) and (**B**) Landsat-8 NDVI (12 July). S: yeast species richness in musts (15Sm) and grapes (15Sg) from 2015; %: yeast frequency as a percentage of the total species (A, *Aureobasidium* spp. in grapes; Hu, *Hanseniaspora uvarum* in musts; M, *Metschnikowia* spp. in musts).

Consequently, Figure 7 presents significant differences in the Sentinel-2 NDVI-S_musts_ correlation (r_average_ = 0.6848). The average r-value was 0.4903 using Landsat-8 information and no significant differences were found; however, the *p*-value was 0.0751, that is, approaching significance. In addition, NDVI and S on the surface of the grapes showed no correlation with either Landsat-8 or Sentinel-2. Furthermore, as expected, significant differences with a high correlation were found between the species richness of musts and grapes and between the NDVI of both satellites. In addition, the yeast species richness of organic samples tends to be more correlated with NDVI than the conventional samples. Likewise, a linear regression analysis between the NDVI, the species richness, and the frequency of each yeast species was conducted. Figure 8 shows some significant results for both satellites. Further information can be found in the supplementary data (Table S1).

**Figure 9:**
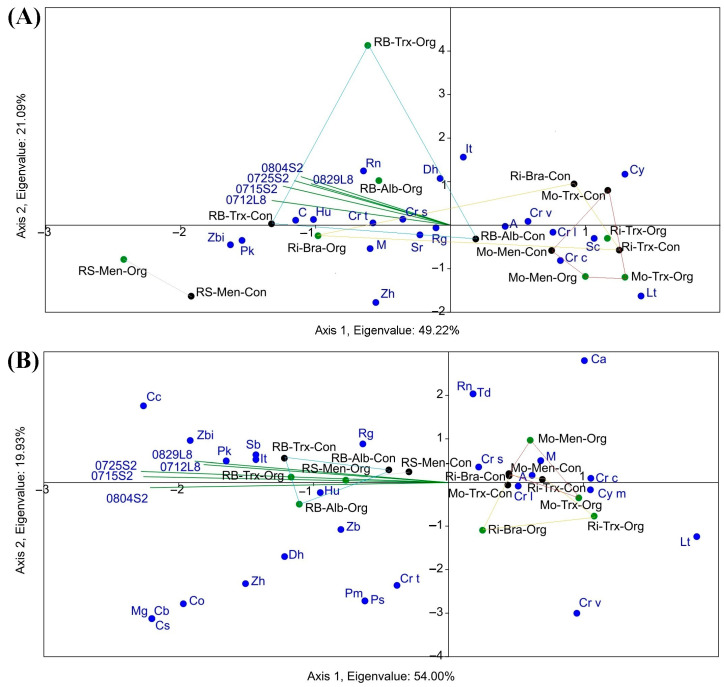
CCA between NDVI calculated using Sentinel-2 (S2) and Landsat-8 (L8) imagery at different dates (0804: 4 August; 0725: 25 July; 0715: 15 July; 0829: 29 August; 0712: 12 July) and regional yeast diversity in grapes (**A**) and musts (**B**) at frequency level (percentage or proportion of species: A, *Aureobasidium* spp.; C, *Candida* spp.; Ca, *Candida apicola*; Cb, *Candida bentonensis*; Cc, *Candida californica*; Co, *Candida oleophila*; Cs, *Candida* cf. *sorbosivorans*; Cr, *Cryptococcus* spp.; Cr c, *Cryptococcus carnescens*; Cr l, *Cryptococcus laurentii*; Cr s, *Cryptococcus stepposus*; Cr t, *Cryptococcus terrestris*; Cr v, *Cryptococcus victoriae*; Cy m, *Cystofilobasidium macerans*; Dh, *Debaryomyces hansenii*; Hu, *Hanseniaspora uvarum*; It, *Issatchenkia terricola*; Lt, *Lachancea thermotolerans*; M, *Metschnikowia* spp.; Mg, *Meyerozyma guilliermondii*; Pk, *Pichia kluyveri*; Pm, *Pichia membranifaciens*; Ps, *Pichia sporocuriosa*; Rg, *Rhodotorula graminis*; Rn, *Rhodotorula nothofagi*; S, *Starmerella bacillaris*; Sc, *Saccharomyces cerevisiae*; Sr, *Sporobolomyces ruberrimus*; Td, *Torulaspora delbrueckii*; Zb, *Zygosaccharomyces bailii*; Zbi, *Zygosaccharomyces bisporus*; Zh, *Zygoascus hellenicus/meyerae*) in the four DOs (Monterrei, Mo; Rías Baixas, RB; Ribeira Sacra, RS; Ribeiro, Ri) according to organic, Org (green) and conventional production system, Con (black), and cultivars (Albariño, Alb; Brancellao, Bra; Mencía, Men; Treixadura, Trx).

Moreover, a CCA and a TB-PLS were performed on both grapes and musts (Figure 8 and Figure 9, respectively) to check NDVI frequency of each yeast species’ correlations in the different DOs.

Regarding the influence of NDVI on the frequency or proportion of the different yeast species, the NDVI values of the triplot of the two satellites in the CCA clustered towards RB and RS for both grapes and musts. However, the CCA showed no correspondence between NDVI and species frequency across varieties and farming systems. Concerning the proportion of species, a higher NDVI value corresponded mainly with the species *H. uvarum*, *Cryptococcus stepposus*, *Cryptococcus terrestris*, *Candida* spp., and *Rhodotorula nothofagi* in grape samples and *Candida californica, H. uvarum, Issatchenkia terricola, Pichia kluyveri, Rhodotorula graminis,* and *Zygosaccharomyces bisporus* in must samples. When species frequency was grouped by genus (CCA not shown), the correspondence of NDVIs was highest with *Hanseniaspora, Candida* and *Rhodotorula* genus in grape samples and *Hanseniaspora, Issatchenkia, Starmerella, Zygoascus* and to a lesser extent with *Debaryomyces, Pichia, Rhodotorula,* and *Zygosaccharomyces* genus in samples of must.

**Figure 10:**
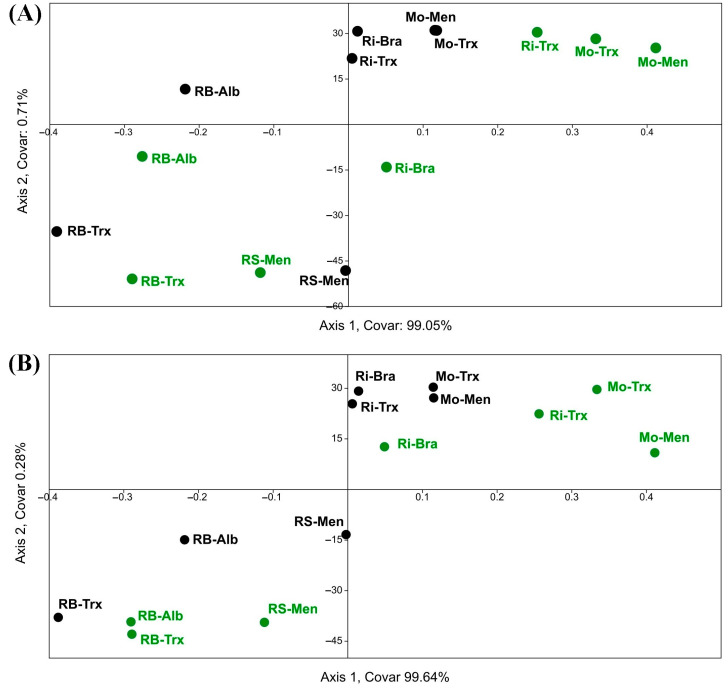
TB-PLS of the correlation between NDVI calculated using Sentinel-2 and Landsat-8 imagery at different dates and regional yeast diversity at frequency level (percentage or proportion of species) in the DOs (Monterrei, Mo; Rías Baixas, RB; Ribeira Sacra, RS; Ribeiro, Ri) according to organic (in green) and conventional production system (in black), and cultivars (Albariño, Alb; Brancellao, Bra; Mencía, Men; Treixadura, Trx) in (**A**) grapes and (**B**) musts.

Moreover, the TB-PLS (Figure 10) correlated NDVI and the DO factor (separating by RB-RS and Mo-Ri DOs in opposite quadrants in both grape and must samples) and partially separated the culture system factor but not the variety factor.

**Figure 11:**
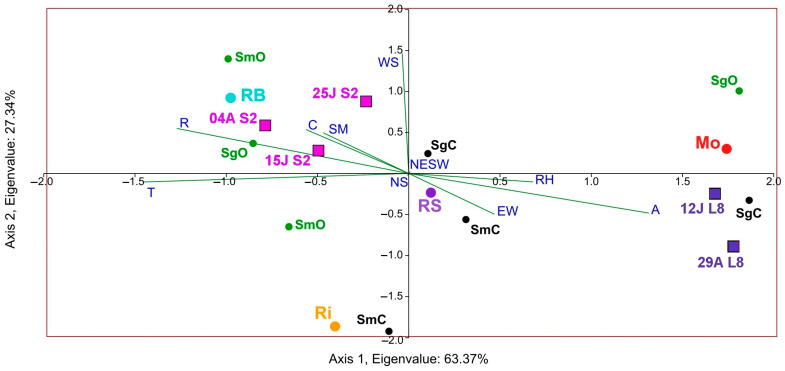
Composite CCA. Impact of factors on the NDVI calculated using Sentinel-2 (S2 in dates 04A: 4 August, 25J: 25 July, and 15J: 15 July) and Landsat-8 (L8 in dates: 29A: 29 August and 12J: 12 July) and species richness in musts (Sm) and grapes (Sg) concerning the DOs (Monterrei, Mo; Rías Baixas, RB; Ribeira Sacra, RS; Ribeiro, Ri) and farming systems (organic (O), in green and conventional (C), in black). Factors: canopy (C), vineyard orientation (EW, NS, NESW), elevation (A), and soil management (SM), and monthly averages of relative humidity (RH), wind speed (WS), rainfall (R), and temperature (T).

Lastly, to better understand the correspondence between satellite NDVI and yeast biogeography, according to species richness in the different DOs and culture systems, a composite CCA was carried out, considering the impact of factors that characterise the microbial terroir and NDVI (Figure 11). Results showed that canopy, rainfall, temperature, and soil management had the highest correspondence with S and NDVI. Furthermore, the triplot showed that the organic samples of the Rías Baixas DO presented higher species richness in both grape and must samples and higher Sentinel-2 NDVI values.

## 4. Discussion

In a previous study carried out with Landsat-8 [16], we found promising results, finding NDVI-S significant correlations over the years (during 2013, 2014, and 2015). However, only Landsat-8 and yeast species richness were employed in that work. Therefore, in the present article, we also employed Sentinel-2 images, with higher resolution compared to Landsat-8. Furthermore, we focused on information related to yeast frequency and yeast biogeographic patterns in musts and grapes obtained in 2015. In this sense, there are significant differences between the two articles. The most important are:

1. In the previous paper, we used data from three years to validate the method (as an experiment to establish a link) by focusing on the terroir and year factors, while in the present document, we focus on the yeast terroir associated with vegetation for each farming system.
2. In the current study, we used more complete one-year data, including other biodiversity data, such as the frequency of each yeast species, and not just the number of species.
3. In the previous work, we used a satellite with a lower resolution (Landsat-8), and it was hypothesised that a higher resolution could improve detection. Therefore, in the current work, we also use Sentinel 2 (to compare them and observe the influence of resolution, including close non-identical dates, which extends the validation of the method).
4. The cultivation system was not explored in depth in the previous article; therefore, it was established as an important factor of study in the present paper. Castrillo et al. [18] found that diversity was lower in grapes than in musts and, in turn, was generally lower in conventional than in organic management, samples of grapes (microbiota present on the surface of the berries), as well as musts for both farming systems.
5. Furthermore, in the present work, we performed linear regressions between NDVI calculated from Landsat-8 and Sentinel-2 images and isolated yeast strains (Figure 8 and supplementary data) to assess the relationship between satellite imagery and yeasts both in number and frequency.

In this paper, at first glance, the NDVI values of Sentinel-2 were higher than those calculated for Landsat-8. However, it should be noted that in all statistical tests a biogeographic relationship or correspondence was observed in the separation of the NDVI values of both satellites with two distinct pairwise between the RB-RS and Ri-Mo DOs, which was in line with what was detected for previous years [16]. In addition, the same NDVI differentiation between the four DOs occurred concerning yeast diversity [18,20]. Furthermore, the satellite NDVI in the PCA biplot (Figure 5) pointed to the DOs with a higher S (RB and RS, which showed slightly lower yeast diversity than the Rías Baixas DO), confirming NDVI-yeast diversity correspondence. However, the biplot for all dates corresponding to Sentinel-2 was mainly directed towards the Rías Baixas DO; meanwhile, the biplot of the NDVI values calculated for the Landsat-8 dates was directed towards the Ribeira Sacra DO. The origin of this difference could be the variation in satellite NDVI values since Sentinel-2 NDVI values were higher than Landsat-8 NDVI values. On the contrary, Ribeiro and Monterrei DOs, which showed lower species richness and NDVI, are in the opposite quadrants. These findings confirm the results obtained during three years for Landsat-8 [16], where higher NDVI implies higher species richness and adds relevance to the fact that the date factor is not a strong factor, regardless of the satellite.

Consequently, Pearson’s coefficient established between Sentinel-2 NDVI and S_musts_ the existence of a positive, moderate-high linear correlation (r = 0.6848, *p* = 0.0069; Figure 7) which was higher than the one obtained using Landsat-8, below the *p* = 0.05 significance level (r = 0.4903, *p* = 0.0751). This result suggests that the ability of Sentinel-2 to assess yeast species richness, which is related to vegetation, is higher than the ability of Landsat-8, which may be due to the higher resolution of the Sentinel-2 satellite. These findings align with [28], who found that VIs generated by Sentinel-2 were more accurate than those from Landsat-8. Similarly, the lack of correlation of any of the NDVIs of the two satellites to the species richness in grape samples could be due to the lower species richness in grape berries compared to musts in the early stages of veraison [29]. Likewise, the higher species richness found in the organic samples had a higher correlation. However, in winemaking, the richness of indigenous yeast strains present in musts is more relevant than in grapes, especially those with fermentative or weakly fermentative capacity, since they are the ones that generate the regional differentiation and the unique character of the wines at the local level [19,30,31,32,33]. Nevertheless, it is worth noting that the species richness value in grapes is strongly correlated with the species richness in musts (r = 0.75); in addition, the NDVIs of both satellites are also strongly correlated (Figure 7).

Regarding the partial correspondence between NDVI and cropping systems (Figure 5 to Figure 11), PCA, CCA, and PLS separated to some extent the organic samples from the conventional ones. In any case, it could not separate the variety factor at all. Furthermore, the biplots and triplots pointed mainly to the organic samples with the highest diversity. This finding is consistent with other works [34,35,36,37], and aligns with previous results [18], where the factor DO (yeast terroir) is stronger than the farming system and much stronger than the variety, which did not influence the separation.

In addition, the CCAs clearly showed that RB and RS had the highest S and NDVI (Figure 6 and Figure 9). The graphic-statistical representation of these two DOs in the CCAs presents reduced areas and a more significant accumulation of yeast species, meaning a greater abundance of species. Moreover, most of the species richness related to NDVI clustered around the DO Ribeira Sacra and Rías Baixas, and are weakly fermentative (Figure 6), i.e., responsible for providing greater complexity and quantity of desirable and specific aromatic compounds to the wines [29,31]. On the contrary, the non-fermentative species are mostly linked to the DOs Ribeiro and Monterrei, the DOs with the lowest NDVI values. Overall, regions with higher NDVI had a ratio of 1.5 more species in both grapes and musts. However, this was not detected in the case of the correspondence between NDVI and species frequency in musts and even less in grapes (Figure 9). Furthermore, the CCA was also unable to identify a correspondence between the highest NDVI values and the yeast species that occurred in the highest proportion, according to Castrillo et al., 2019 [18]. The highest NDVI corresponds with *H. uvarum*, as well as with *Starm. bacillaris,* but not with *Aureobasidium* spp., *Cryptococcus,* and *Metschnikowia* spp. While *Starm. bacillaris, Pichia* spp. and even *H. uvarum* (one of the most widely distributed yeasts present worldwide) have been proposed as non-*Saccharomyces* that provide differentiation to quality wines [38,39,40,41], other species such as *Cryptococcus* spp. or *Aureobasidium* spp., have no oenological potential. *Metschnikowia* spp. or *L. thermotolerans,* which are also valuable non-*Saccharomyces* yeasts with oenological potential [42,43], are opposed to the NDVI biplot even if present in high proportion in musts.

Other authors have associated the diversity of yeast species with particular conditions such as territories, management of cultivation systems, and soil, environmental, and other factors without anthropogenic influence [44,45,46,47]. The fact that not only the separation between DOs but also the PCA and CCA clustered the Mo-Ri and RB-RS pairs confirms the presence of biogeographical separation, and it is consistent with a previous study carried out for the frequency of species [18]. Moreover, it is worth noting that the correspondence between NDVI and yeast species (Figure 6 and Figure 9) partially coincides with the biogeographical patterns found for the different DOs in that study. Furthermore, the area of yeast concentration for the different DOs is less in proportion in the must samples than in the grape samples, which is consistent with Pearson’s r results. However, we consider that this differentiation is due to the DO factor since no correlation is observed between NDVI values and biogeographic patterns of yeasts at the species proportion level, so the results are not conclusive to predict the proportion of species from the NDVI obtained in each region. This result shows that species richness is influenced by vegetation (NDVI), while the proportion of species of the different biogeographic patterns is more influenced by the DO factor, which in turn includes many other added factors. We suggest that more vegetation can generate greater species richness due to the microclimate associated with the vine phyllosphere, for example, by better preserving favourable temperature and humidity conditions. Other authors have also stated that vegetation can influence the associated microbiota [48]. However, non-physical factors such as genetic isolation, and other factors such as soil, rhizosphere-associated microbiota, climatic changes, animal vectors, nearby native forests, and cultural practices, define biogeographic patterns beyond the canopy [49,50].

Finally, the results found for the influence of the factors canopy, rainfall, temperature, organic cultures system, and soil management align with the findings for the three years with Landsat-8 [16]. In this work, they had the highest correspondence with Sentinel-2 NDVI and species richness in both grapes and musts samples. However, it is worth mentioning that the Landsat NDVI triplot was directed towards the conventional samples (with lower species diversity than the organic samples). Compared to Sentinel-2, the data are oppositely separated in the graph, demonstrating the marked difference in the higher resolution or ability of Sentinel-2 to correlate NDVI with yeast diversity. Moreover, Figure 3 shows the correlation between elevation and annual accumulated rainfall, in which several factors, such as proximity to the sea or climatic influence, play a role.

The concept of terroir establishes that the plant and the environment in which the plant grows, with its climate variations, crop features, and viticultural and oenological techniques, interact in a certain way that cannot be replicated somewhere else, providing a site-specific and exclusive quality to the cultivation place [3]. Furthermore, terroir also affects the diversity of yeasts, linking them to a geographical area [15,34]. Therefore, the relationship between differentiated wine quality and indigenous yeasts is well-known as one of its factors [15,51,52]. In fact, the relationship between vegetation, yeasts and wine distinction can be affected at several levels: On the one hand, the amount of vegetation affects parameters related to plant physiology and the chemical compounds it generates, such as the concentration of sugars and grape acidity, which in turn influence yeast biodiversity [29,53]. On the other hand, the plant environment and the canopy structure affect the microclimate of the crop, such as temperature, solar radiation, or relative humidity, which are determinants for yeast development. Therefore, factors that directly or indirectly affect vegetation growth, such as rainfall, irrigation, fog, soil, or altitude, also influence yeast growth [29,44,54,55,56]. Moreover, we obtained better accuracy using Sentinel-2 images, which could confirm the correlation of NDVI with yeast species richness since Sentinel-2 is more accurate than Landsat-8 in identifying vegetative development, probably due to the higher resolution [28]. Additionally, the correlation between the different dates of Sentinel-2 and Landsat-8 images was very high (r > 0.94 and 0.99 respectively), showing that the amount of vegetation remained stable during the study period, and that vegetation development had already stopped, or almost stopped. Therefore, the images from each satellite were taken as close as possible to the phenological stage of veraison, as this is a critical moment in the vineyard and is often used as a reference in viticulture [9], showing the strongest correlations (Figure 8). Although due to their complexity, the microbial data for each sample are not large enough to make predictions, the regression analyses showed some correlation for some species such as *Aureobasidium* spp., *H. uvarum*, and *Metschnikowia* spp. This fact could be explained because the first two species are the most commonly present in grapes and musts, respectively [29]. Moreover, the case of *Aureobasidium* is very interesting because the correlation with satellite NDVI is negative, i.e., the more vegetation the lower the percentage of these species. This trend was also found for *Metschnikowia* spp., a yeast with great oenological interest [43]. More detailed studies confirming these results could be useful for winemakers so that a specific leaf removal could affect the frequency of desired species in the production of distinctive wines, such as those of natural fermentation or organic production.

The results presented in this work are exciting because they could lead to a new research line allowing microbial terroir characterisation using remote sensing. Different microbial species provide different complexity and aromatic compounds to the wines [57,58,59], and as in previous studies, the findings are consistent with the biogeographic patterns of terroir-associated yeasts inherent in the satellite NDVI. Therefore, spectral information such as vegetation indices could be useful to define different areas related to microbial terroir. Moreover, since NDVI represents the development of the vineyard within each pixel area and is closely related to the amount of vegetation [60], these results confirm the terroir results from many variables, such as vineyard management, landscape, climate, or plant genetics. Furthermore, the results suggest that although each terroir component may be similar when comparing two particular DOs, each component contributes to the overall result, making it possible for satellite NDVI to detect changes in areas with different terroirs and, therefore, in yeasts. For example, Ribeiro and Ribeira Sacra have similar rainfall and altitudes, yet only the latter showed the highest species richness values along with Rias Baixas. Additionally, NDVI could help detect variations in some of its components, such as yeasts, without the need to define each component individually, allowing the use of tools such as remote sensing for the delimitation of DO.

Future research could include the analysis of ultrahigh-resolution imagery, such as the one provided by UAVs, since Sentinel-2 achieved higher correlations than Landsat-8, probably due to the higher resolution. Moreover, higher resolution imagery or longer historical data in other DOs, including more years, could help refine the technique as a tool for assessing microbial diversity or mapping terroir at the local or plot level for the winemaker, not just at the regional DO level.

## 5. Conclusions

This paper demonstrates the possibility of using NDVI calculated from high-resolution multispectral images from Sentinel-2 and Landast-8 to assess yeasts in the vineyard. Yeast diversity is affected by vegetation and NDVI is able to detect it. The global results of this study confirmed that:

Free and high-resolution satellite multispectral imagery can establish differences in yeast terroir according to NDVI.

The species richness is influenced by vegetation, whereas the proportion of species of the different biogeographic patterns is more influenced by the Designation of Origin factor, which in turn includes many other factors linked to each other.

The moderate positive correlation found between NDVI and yeast species richness in both must and grape surface samples, using Landsat-8 and Sentinel-2 satellite images acquired at various dates close to veraison, gives stability to the method and supports the idea of developing a hypothetical predictive model in the future that is able to estimate yeast species richness from NDVI.

Sentinel-2 detected a higher NDVI-yeast diversity correlation than Landsat-8 as well as better delimitation of Designations of Origin at the yeast terroir level, suggesting that better satellite resolution improves the ability to assess yeast species richness.

Regarding sample type and farming system, musts achieved higher values than grapes in the linear regressions between vegetation and yeast diversity (species richness and frequency), with a tendency to have a higher correlation in organic samples. This is consistent with the results that musts (vs grapes) and organic (vs conventional) pairwise samples had the highest yeast biodiversity indices.

Moreover, a higher proportion of fermentative or weakly fermentative yeast species was found in grapes, and musts corresponded to the DOs with the highest NDVI values. However, NDVI values did not correlate well with biogeographic patterns since higher vegetation did not always correspond with the yeast species found with a higher proportion (percentage of each species) in each Designation of Origin.

The relationship found in this study between yeasts and NDVI is valuable because it implies that remote-sensed NDVI could be employed as a model or factor for wine differentiation and characterisation. The results are promising and could lead to a new research line allowing microbial terroir characterisation using remote sensing. It is, therefore, interesting to address these aspects through innovative techniques. To this end, it would be necessary to perform studies developed explicitly for this purpose, desirably including higher resolution imagery, such as the one obtained using airborne or drones, aiming to better identify the relationship between the spectral information and the yeast diversity.

## Figures and Tables

**Table 1:** Bray-Curtis PERMANOVA between factors and Sentinel-2 NDVI: (A) Designations of Origin (DOs: Monterrei, Mo; Rías Baixas, RB; Ribeira Sacra, RS; Ribeiro, Ri)–culture system set (Organic, Org; Conventional, Con); (B) DOs; (C) grape varieties (Albariño, Alb; Brancellao, Bra; Mencía, Men; Treixadura, Trx); and (D) cultivation system.

(A) DO-Org/Con			Mo-Con	Mo-Org	RB-Con	RB-Org	Ri-Con	Ri-Org	RS-Con	RS-Org
**W-g_s.o.s._** **:**	0.0316	**Mo-Con**		0.3367	0.3334	0.3313	0.3339	1	0.3382	0.3356
**F:**	*11.00*	**Mo-Org**	37.54		0.3270	0.3360	0.3184	0.3271	0.3359	0.3319
**p_(same)_:**	*0.0029*	**RB-Con**	40.01	*71.67*		1	0.3329	0.3337	0.3322	0.3310
		**RB-Org**	*121.00*	*104.40*	0.22		0.3345	0.3310	0.3315	0.3286
		**Ri-Con**	27.84	58.20	23.22	*83.29*		0.3397	0.3299	0.3331
		**Ri-Org**	0.27	3.89	6.60	7.83	1.29		0.6627	0.6708
		**RS-Con**	16.77	21.10	6.81	25.96	3.95	0.53		1
		**RS-Org**	44.94	26.65	3.30	13.52	45.15	0.98	0.98	
**(B) DOs**			**Mo**		**RB**		**Ri**		**RS**	
**W-g_s.o.s._** **:**	0.1314	**Mo**			*0.0279*		0.1737		0.1340	
**F:**	*7.76*	**RB**	*18.40*				*0.0275*		0.0654	
**p_(same)_:**	*0.0046*	**Ri**	2.05		*16.81*				0.3275	
		**RS**	3.55		*22.16*		1.34			
**(C) Grape varieties**			**Men**		**Trx**		**Alb**		**Bra**	
**W-g_s.o.s._** **:**	0.1314	**Men**			0.7486		0.1311		0.7985	
**F:**	0.96	**Trx**	0.17				0.3231		0.8200	
**p_(same)_:**	0.4355	**Alb**	2.82		1.78				0.3364	
		**Bra**	0.35		0.13		*24.96*			
**(D) Org-Con**			**Org**				**Con**			
**W-g_s.o.s._:** **F:** **p_(same)_:**	0.3964 1.23 0.2777	**Org**					0.2855			
		**Con**	1.231							

Permutation (N) = 9999 and total sum of squares = 0.437 in all tests; p_(same)_: p_total_ (between groups); W-g_s.o.s._: within-group sum of squares. Below the diagonal, the statistical F-values are given. Above the diagonal, the *p*-values (significance level) are given. Relevant values (F) and significance (*p* < 0.05) are highlighted in italics.

## Data Availability

The data presented in this study are openly available at DOI: https://doi.org/10.20870/oeno-one.2019.53.3.2379 and DOI: https://doi.org/10.20870/oeno-one.2022.56.4.4898.

## Supplementary Materials

The following supporting information can be downloaded at: https://www.mdpi.com/article/10.3390/s23042059/s1, Table S1. Correlation coefficients between NDVI values calculated from Sentinel 2 and Landsat 8, yeast frequency, and yeast species richness (S). Year 2015.
