# Supplementary material for: Can Satellite Remote Sensing Assist in the Characterization of Yeasts Related to Biogeographical Origin?
Source: Sensors (Basel). 2023 Feb 11;23(4):2059. doi: 10.3390/s23042059 (PMC9962804; doi:10.3390/s23042059)
Supplement: Supplementary file 1 [file sensors-23-02059-s001.zip › sensors-2191562-supplementary.pdf]

sensors

MDPI

Article

Can satellite remote sensing assist in the characterization of yeasts related to biogeographical origin?

David Castrillo [1,*] Pilar Blanco [1] and Sergio Vélez [2,*]

1 Estación de Viticultura e Enoloxía de Galicia (EVEGA-AGACAL), Ponte San Clodio s/n, 32428 Leiro-Ourense, Spain
2 Information Technology Group, Wageningen University & Research, 6708 PB Wageningen, Netherlands
* Correspondence: david.castrillo.cachon@xunta.gal

Supplementary material

Table S1. Correlation coefficients between NDVI values calculated from Sentinel 2 and Landsat 8, yeast frequency, and yeast species richness (S). Year 2015. Levels of statistical significance (Sig.): ns, non-significant; *, $p < 0.05$. Data with different letters indicate significant differences.

	NDVI date	Sentinel 2						Landsat 8			
Parameter		15 July		25 July		4 August		12 July		29 August	
Grapes (frequency)	A	-0.56	*	-0.52	*	-0.51	*	-0.62	*	-0.50	*
	C	0.21	ns	0.20	ns	0.22	ns	0.49	*	0.37	*
	Cr	-0.17	ns	-0.17	ns	-0.14	ns	-0.23	ns	-0.31	*
	Cm	-0.16	ns	-0.10	ns	-0.06	ns	-0.25	ns	-0.10	ns
	Dh	0.36	*	0.38	*	0.41	*	0.31	*	0.42	*
	Hu	0.65	*	0.62	*	0.58	*	0.68	*	0.62	*
	It	0.30	ns	0.33	*	0.35	*	0.32	*	0.44	*
	Lt	-0.44	*	-0.47	*	-0.50	*	-0.49	*	-0.50	*
	M	0.22	ns	0.15	ns	0.10	ns	0.37	*	0.17	ns
	P	0.12	ns	0.10	ns	0.11	ns	0.40	*	0.23	ns
	R	0.24	ns	0.24	ns	0.21	ns	0.14	ns	0.19	ns
	Sc	-0.36	*	-0.34	*	-0.39	*	-0.42	*	-0.32	*
	S	-0.07	ns	-0.07	ns	-0.12	ns	0.06	ns	0.05	ns
	Zh	0.06	ns	-0.01	ns	-0.04	ns	0.08	ns	-0.06	ns
	Zb	0.11	ns	0.08	ns	0.09	ns	0.39	*	0.21	ns
Musts (frequency)	A	-0.49	*	-0.48	*	-0.47	*	-0.43	*	-0.39	*
	C	0.06	ns	0.05	ns	0.02	ns	-0.16	ns	-0.13	
	Sb	0.67	*	0.68	*	0.65	*	0.60	*	0.69	*
	Cr	-0.22	ns	-0.20	ns	-0.15	ns	-0.15	ns	-0.20	ns
	Cy	-0.05	ns	-0.05	ns	-0.03	ns	-0.04	ns	0.02	ns
	Dh	0.50	*	0.52	*	0.57	*	0.11	ns	0.19	ns
	Hu	0.72	*	0.69	*	0.68	*	0.58	*	0.52	*

	**It**	0.53	*	0.50	*	0.45	*	0.69	*	0.62	*
	**Lt**	-0.58	*	-0.57	*	-0.54	*	-0.37	*	-0.42	*
	**M**	-0.64	*	-0.63	*	-0.65	*	-0.64	*	-0.63	*
	**Mg**	0.40	*	0.41	*	0.47	*	-0.14	ns	-0.09	ns
	**P**	0.56	*	0.57	*	0.53	*	0.76	*	0.74	*
	**R**	0.23	ns	0.19	ns	0.16	ns	0.21	ns	0.16	ns
	**Td**	0.06	ns	-0.01	ns	-0.04	ns	0.08	ns	-0.06	ns
	**Zh**	0.53	*	0.56	*	0.63	*	0.15	ns	0.23	ns
	**Z**	0.74	*	0.75	*	0.72	*	0.57	*	0.68	*
**S**	**SmC**	0.62	*	0.52	*	0.51	*	0.87	*	0.63	*
	**SmC**	0.77	*	0.73	*	0.70	*	0.75	*	0.74	*
	**SmO**	0.95	*	0.92	*	0.91	*	0.95	*	0.95	*
	**SmO**	0.91	*	0.88	*	0.86	*	0.84	*	0.89	*
	**SgO**	0.96	*	0.96	*	0.95	*	0.82	*	0.95	*
	**SgC**	0.83	*	0.82	*	0.79	*	0.67	*	0.79	*
	**SgO**	0.13	ns	0.11	ns	0.06	ns	0.07	ns	0.08	ns
	**SgC**	-0.03	ns	-0.10	ns	-0.14	ns	0.13	ns	-0.06	ns
